# Mild encephalitis/encephalopathy with reversible splenial lesion (MERS) associated with respiratory syncytial virus and *Pseudomonas putida* infection: A case report

**DOI:** 10.1016/j.heliyon.2024.e39685

**Published:** 2024-10-22

**Authors:** Yanxiang Chen, Kai Dai, Bofu Ruan, Hui Wang, Guonan Zhou, Ying Jiang

**Affiliations:** a Department of Neurology, Xiaolan People's Hospital of ZhongShan (The Fifth People's Hospital of ZhongShan), 65#, Middle Section of Jucheng Avenue, Xiaolan, Zhongshan, Guangdong Province, 528400, China; b Department of Neurology, The Third Affiliated Hospital of Sun Yat-sen University, 600# Tianhe Road, Guangzhou, Guangdong Province, 510630, China; c Department of Encephalopathy, Zhongshan Chenxinghai Hospital of Integrated Traditional Chinese and Western Medicine, 18# Zhuyuan Road, Zhongshan, Guangdong Province, 528400, China

**Keywords:** Encephalitis, *Pseudomonas putida*, Respiratory syncytial virus, Reversible splenial lesion

## Abstract

**Background:**

Clinically mild encephalitis/encephalopathy with a reversible splenial lesion (MERS) is a mild encephalopathy, which may be associated with various pathogens, including virus and bacteria. However, there have been no reports on MERS associated with co-infection by respiratory syncytial virus (RSV) or *Pseudomonas putida* in adults.

**Case presentation:**

We reported a 29-year-old Chinese woman with MERS associated with RSV and *Pseudomonas putida*. This woman presented with fever, sore throat, cough, and altered mental states. The results of RSV-RNA in the specimens from throat and sputum culture of *Pseudomonas putida* were positive. The initial head CT scan on the day of admission revealed abnormal hypodense lesions in the suprasellar cistern (SCC). Subsequent brain magnetic resonance imaging (MRI) also demonstrated abnormal hypersignals in the same region. The patient's altered mental status improved on day after ceftriaxone and low-dose corticosteroid therapy. The SCC hypersignal on MRI completely resolved after three weeks, and no recurrence of symptoms occurred during the two-month follow-up period.

**Conclusions:**

It is the first reported case of MERS associated with RSV and *Pseudomonas putida* in the adult, which broadens the spectrum of potential etiologies in MERS. When a patient with a respiratory tract infection presents with neurological symptoms, the possibility of MERS should be considered.

## 1 Introduction

Mild encephalitis/encephalopathy with a reversible splenial lesion (MERS) is a clinical syndrome distinguished by transient neurological manifestations that correlate with reversible splenial lesions in the corpus callosum, as evidenced by magnetic resonance imaging (MRI) [1]. Common neurological symptoms include disorders of consciousness, delirium, and seizures, with additional symptoms such as fever, headache, drowsiness, ataxia, dysarthria, nausea, and blurred vision, all of which typically resolve within one month. The etiology of MERS is diverse, including non-infectious and infectious causes. Non-infectious conditions comprise metabolic disturbances [2] and inflammatory processes within the central nervous system (CNS) [3]. Infectious etiologies comprise a series of viral agents, such as influenza virus, mumps virus, varicella-zoster virus, adenovirus [1,4], and recently identified SARS-CoV-2 associated with COVID-19 [5]. In MERS cases, bacterial infections are rarely identified as causative agents. Moreover, there are no reports on MERS associated with co-infection by virus and bacteria. Herein, we present the first case of MERS associated with a dual infection involving respiratory syncytial virus (RSV) and *Pseudomonas putida* in an adult patient.

## 2 Case presentation

A 29-year-old Chinese woman who was a nurse working in a hospital had a severe respiratory infection with fever, headache, sore throat, and coughing for six days. She has been suffering from insomnia for a week, due to taking care for her son who was hospitalized for adenovirus infection. At first, she was sent to the Traditional Chinese Medicine department due to fever, and then transferred to the inpatient unit of neurology the next day due to mental abnormality, presenting with gibberish, dull expression, alternating silence and agitation, disorientation in time and space. The Glasgow Coma Score was 12 (E4V3M5; eye opening, 4; verbal response, 3; motor response, 5), with a body temperature of 38.5 °C, blood pressure of 134/85 mmHg, pulse rate of 112 beats/min, respiratory rate of 22 breaths/min, and SpO2 of 96 % on room air. Lung auscultation revealed clear breath sounds without any significant rales or rhonchi. Neurological examination was positive for nuchal rigidity. However, Kernig's and Brudzinski's signs were negative. In addition, other focal neurologic signs were negative. She had no history of allergies or any specific family history.

Blood routine displayed normal white blood cells, 7.44 10^9/L (normal range 3.5–9.5 10^9/L). The serum electrolytes showed a moderate hypokalemia with 2.95 mmol/L (normal range 3.5–5.3 mmol/L) and increased inflammatory markers with C-reactive protein, 76.3 mg/L (normal range 0–6 mg/L), interleukin 6, 44.40 pg/mL (normal range 0–7.0 pg/mL), procalcitonin, 0.414 ng/mL (normal range 0–0.05 ng/mL), and serum amyloid A (SAA), 185.6 mg/L (normal rang 0–10 mg/L). Creatinine and liver function tests were normal. Autoimmune disease related examination showed triiodothyronine (T3) with 0.90 nmol/L (normal rang 1.3–3.1 nmol/L), free triiodothyronine (FT3) with 2.74 pmol/L (normal range 3.1–6.8 pmol/L), and negative rheumatism immune antibodies. The etiological agent of COVID-19, SARS-CoV-2 was not detected. The Dengue virus NS1 antigen (NS1-Ag) was also absent in the serum. Additionally, Mycoplasma pneumoniae immunoglobulin M (IgM) was not detected through serum testing. The results of blood culture and sputum analysis for *Mycobacterium tuberculosis* were negative. However, the respiratory syncytial virus RNA (RSV-RNA) was identified via polymerase chain reaction (PCR) from a throat swab specimen. On the fourth day post-collection, a sputum culture revealed a positive result for *Pseudomonas putida*.

**Fig. 1:**
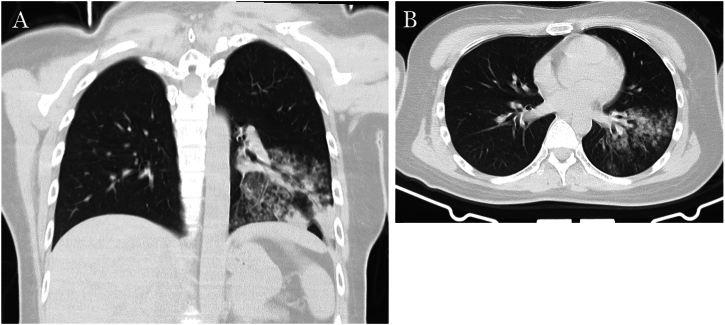
Chest CT on admission showed lung pneumonia in the left inferior lobe (A, B).

**Fig. 2:**
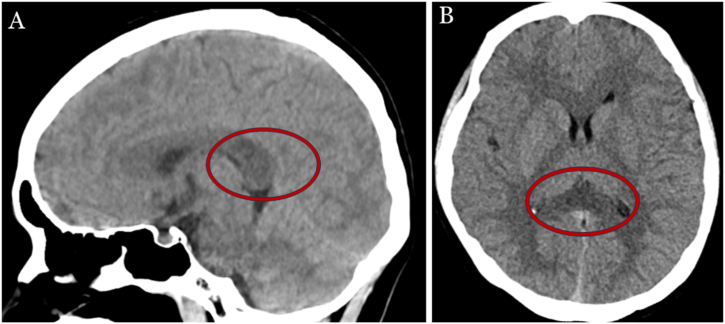
Head CT on admission showed abnormal hypodensity lesions in the SCC (A, B).

**Fig. 3:**
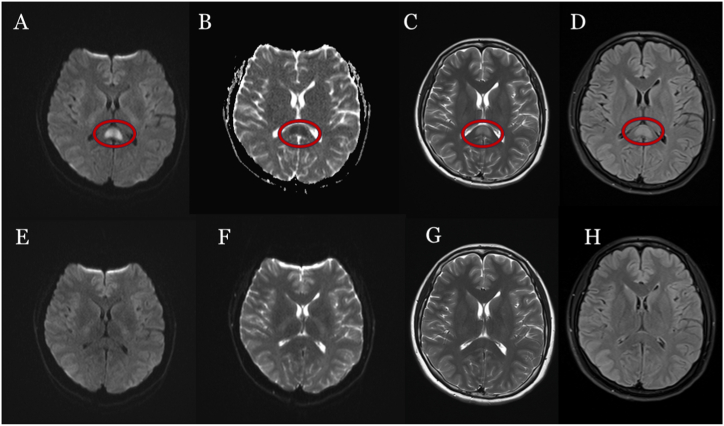
Brain MRI on admission and after treatment. (A–H) MR imaging reveals the SCC as hypersignal on diffusion-weighted imaging (DWI) (A) with a low apparent diffusion coefficient (ADC) value (B), and exhibiting high signal intensity on T2-weighted images (C) as well as on FLAIR images (D). Abnormalities detected on MRI completely resolved after 23 days (E–H).

A lumbar puncture was conducted on the second day after admission. Given the patient's clinical presentation suggestive of a CNS infection, we initiated empirical treatment to provide broad-spectrum coverage while awaiting further diagnostic confirmation. The cerebrospinal fluid (CSF) exhibited a transparent appearance, with an opening pressure of 160 mmH_2_O (normal range 80–180 mmH_2_O). A comprehensive laboratory analysis of the CSF showed WBC count of 2 × 10^6/L (normal range 0–8 cells/μL), protein content of 284.44 mg/L (normal range 200–400 mg/L), chloride of 117.00 mmol/L (normal range 120–132 mmol/L), and glucose of 6.06 mmol/L (normal range 2.22–3.89 mmol/L). On the third day after admission, the results of metagenomic next-generation sequencing (mNGS) of the CSF samples testing for pathogenic DNA and RNA were negative. The autoimmune encephalitis panel, including a spectrum of antibodies such as NMDAR, AMPAR, GABABR, GAD65, DPPX, IgLON5, GlyR1, mGluR5, CASPR2, AQP4, MOG, GFAP, and MBP, was negative. However, the presence of oligoclonal bands (OCB) was identified in both CSF and blood samples. The electroencephalogram (EEG) was normal and showed no epileptiform activity. Chest computed tomography (CT) revealed left lung pneumonia, with significant involvement of the inferior lobe (see Fig. 1A and B), and head CT revealed abnormal hypodensity lesions in the SCC (see Fig. 2A and B) on the admission day. Subsequent brain MRI revealed an abnormal hypersignal in the SCC on diffusion-weighted imaging (DWI) and hypodensity signals on apparent diffusion coefficient (ADC). The same area also showed slight hypersignal on T2-weighted imaging (T2WI) and fluid-attenuated inversion recovery (FLAIR) imaging (see Fig. 3A–D). MR enhancement and MR angiography did not show obvious abnormalities. On admission, considering the patient's symptoms and the preliminary diagnosis of possible CNS infection, we initiated a multifaceted treatment approach. We administered dexamethasone (10 mg/day), and then gradually tapered over during the 13-day hospitalization. Ceftriaxone (4 g/day) was given for 10 days, and acyclovir (500 mg, three times a day) was given for 13 days from the day of admission. The patient's altered mental status improved and meningeal irritation signs turned to negative two days after admission. The mental state of the patient was normal on the fourth day after admission and she was discharged 13 days after hospitalization. SCC hypersignal on MRI completely resolved after three weeks (Fig. 3E–H), and no recurrence of symptoms occurred during the two-month follow-up period.

## 3 Discussion

It is the first reported case of MERS associated with RSV and *Pseudomonas putida* in the adult. Brain MRI demonstrated abnormal hyperintense signals in the SCC. The altered mental status of patient improved on the second day after antibiotic and low-dose corticosteroid therapy. The SCC hyperintensity on MRI completely resolved after three weeks, and no recurrence of symptoms occurred during the two-month follow-up period.

In 2004, Tada et al. [1] first described the clinical characteristics of 15 patients who had encephalitis/encephalopathy with a reversible lesion in the SCC, referring to this condition as MERS. Most cases of MERS have been reported in the pediatric population. The most commonly associated pathogen is the influenza virus (34 %), followed by rotavirus (12 %) and mumps virus (4 %) [7]. Our case expands the clinical spectrum of MERS by identifying a dual infection with RSV and *Pseudomonas putida* as potential triggers in an adult patient, which emphasizes the diversity of infectious etiologies that may lead to MERS and the necessity of comprehensive diagnostic evaluations.

Common neurological manifestations include delirium (54 %), altered consciousness (35 %), and seizures (33 %) [6]. In child-onset MERS, the prodromal manifestation of MERS was non-specific [7]. In most patients, neurological symptoms were preceded by prodromal symptoms (88 %), primarily fever (78 %), headache (50 %), seizures (22 %), and altered consciousness (22 %) [8]. In this current case, the patient presented with symptoms including persistent severe respiratory infection with fever, headache, sore throat and coughing, progressive consciousness disturbance, delirium, and positive meningeal irritation signs. The co-infection of RSV and *Pseudomonas putida* presents with a complex clinical picture, and the contribution of each pathogen to the neurological manifestations remains to be elucidated.

RSV has been found to cause neurological complications, with more frequent reports in the pediatric population [9,10]. The clinical manifestations include seizures, encephalopathy, and encephalitis [11]. The possible mechanisms of RSV causing neurological complications are as follows: RSV can access the CNS through various routes, including hematogenous dissemination, direct infection of neuronal cells, retrograde axonal transport along peripheral nerves, disruption of the blood-brain barrier, and a “Trojan horse” mechanism utilizing infected immune cells [12]. These diverse pathways allow the virus to infiltrate and potentially induce damage to the CNS, though the precise underlying mechanisms require further investigation. *Pseudomonas putida*, a Gram-negative bacterium known for its environmental adaptability and opportunistic pathogenicity, particularly affects immunocompromised individuals. As a member of the fluorescent Pseudomonas species, it exhibits metabolic diversity and ecological flexibility, allowing it to survive in diverse settings such as soil, fresh water, and damp hospital environments [13]. This bacterium has the capacity to colonize the respiratory tract, often leading to pulmonary infections in patients with pre-existing health conditions like cancer, liver cirrhosis, or post-transplantation states [14]. The virulence factors produced by *Pseudomonas putida*, coupled with its resistance to host immune responses, facilitate the development of nosocomial pneumonia, frequently associated with mechanical ventilation and other invasive respiratory devices [15]. Additionally, *Pseudomonas putida* can cause rare but severe cranial infections, including ventriculitis associated with the use of external ventricular drains (EVDs) [16]. The pathogenesis of such infections is complex, involving the bacterium's biofilm formation on device surfaces, antimicrobial resistance, and the host's impaired immune status. In our case, it is considered that the patient is in a state of weakened immunity due to insomnia and fatigue. However, one limitation of our case is the uncertainty of the exact role of *Pseudomonas putida* in the pathogenesis of MERS. The presence of these two distinct pathogens in our patient raises intriguing questions about potential synergistic or antagonistic interactions between them. We cannot definitively prove the nature of this association, further studies are needed to elucidate the mechanisms underlying such co-infections and their impact on the development of MERS. However, the potential synergistic effect of these pathogens in the etiology of MERS remains unclear. The MRI abnormalities in MERS, which may be caused by cytotoxic edema, have been defined as cytotoxic lesions of the corpus callosum [17]. According to the literature [1,18], the reversible splenial lesions (SCLs) in MERS are thought to be caused by mechanisms including intramyelinic edema due to myelin layer separation, interstitial edema in densely packed axons of the splenium, and transient inflammatory infiltrates resembling those observed in multiple sclerosis. Additionally, cerebral edema from hyponatremia and the influence of antiepileptic drugs (AEDs) on water balance may lead to reversible SCLs. The underlying pathophysiological mechanisms may bear similarities to those observed in high altitude cerebral edema (HACE), suggesting a role for hemodynamic alterations and compromised blood-brain barrier integrity. In our case, we found the remarkable elevation of serum IL-6 at the onset, and OCB also were positive in CSF and blood, which may indicate an immune response in the CNS, including cytokine-mediated processes and blood-brain barrier disruption. However, the exact pathogenic basis for the specific involvement of the SCLs remains to be elucidated.

MERS is a distinctive neuroinflammatory condition that requires differentiation from other CNS disorders, particularly within the spectrum of leukoencephalopathies. The MRI characteristics of MERS, including the reversible splenial lesion, are instrumental in distinguishing it from conditions such as acute disseminated encephalomyelitis (ADEM), reversible posterior leukoencephalopathy syndrome (RPLS), and hereditary leukodystrophies. MERS typically presents with a milder clinical picture compared to ADEM, which often follows a post-infectious or post-vaccination course and exhibits asymmetric T2-hypersignal lesions on MRI, may show post-contrast enhancement. In contrast, MERS lesions are characterized by symmetric involvement of the splenium of the corpus callosum and exhibit restricted diffusion, indicative of cytotoxic edema [1]. In addition, MERS need to be differentiated from other CNS infections by its clinical presentation and laboratory findings. For instance, patients with MERS often have a history of recent viral infection, and their CSF typically shows nonspecific changes, and may even be normal, unlike bacterial or fungal infections which usually present with significant pleocytosis and elevated protein levels [19]. Moreover, certain biomarkers such as elevated interleukin-6 (IL-6) and urinary β2-microglobulin (β2MG) have been observed in patients with MERS, which can further assist in differentiating it from other CNS infections [20]. The reversibility of MERS suggests a more favorable prognosis when compared to other CNS infections, which may have more severe outcomes.

A consensus on the management of MERS has not been established. The possible effectiveness of corticosteroids and immunoglobulins in addressing the elevated cytokine levels detected in CSF remains hypothetical. In typical MERS presentations, the use of methylprednisolone pulse therapy combined with high-dose gamma globulin treatment is not always necessary [6]. The specific treatment approach should be tailored to the individual patient's needs. Our case recovered quickly after early use of low-dose corticosteroid. The prognosis for MERS patients is generally favorable, with complete recovery without sequelae following appropriate treatments within one month [18].

## 4 Conclusion

It is the first reported case of MERS associated with RSV and *Pseudomonas putida* in the adult, which broadens the clinical spectrum of MERS. MERS is a clinical and radiological syndrome with generally favorable prognosis. When a patient with a respiratory tract infection presents with neurological symptoms, the possibility of MERS should be considered.

## CRediT authorship contribution statement

**Yanxiang Chen:** Writing – original draft, Visualization, Data curation, Conceptualization. **Kai Dai:** Writing – original draft, Visualization. **Bofu Ruan:** Writing – original draft, Data curation. **Hui Wang:** Validation, Resources. **Guonan Zhou:** Validation, Resources. **Ying Jiang:** Writing – review & editing, Validation, Supervision.

## Data availability statement

The original details in this study are included in the article; further inquiries can be directed to the corresponding author.

## Consent for publication

Written informed consent was obtained from the patient for the publication of this case and any potentially identifiable images or data included in this article.

## Funding

This research did not receive any specific grant from funding agencies in the public, commercial, or not-for-profit sectors.

## Declaration of competing interest

The authors declare that they have no known competing financial interests or personal relationships that could have appeared to influence the work reported in this paper.
