# Supplementary material for: Mild encephalitis/encephalopathy with reversible splenial lesion (MERS) associated with respiratory syncytial virus and Pseudomonas putida infection: A case report
Source: Heliyon. 2024 Oct 22;10(20):e39685. doi: 10.1016/j.heliyon.2024.e39685 (PMC11538784; doi:10.1016/j.heliyon.2024.e39685)
Supplement: Multimedia component 1 [file mmc1.docx]

In the case report entitled "Mild Encephalitis/Encephalopathy with Reversible Splenial Lesion (MERS) Associated with Respiratory Syncytial Virus and Pseudomonas putida Infection: A Case Report," several items from the CARE Checklist were marked as 'Not Applicable.' for the following reasons:

1. **5d Relevant past interventions with outcomes**: Not applicable as this case represents a novel presentation of MERS associated with dual infection, without prior similar interventions influencing the clinical course.
2. **8b Diagnostic challenges (such as access to testing, financial, or cultural)**: Not applicable due to the absence of significant diagnostic obstacles; diagnostic assessments were conducted without hindrance.
3. **8d Prognosis (such as staging in oncology) where applicable**: Not applicable as the report focuses on an acute neurological event rather than a chronic condition requiring staging, such as in oncology.
4. **10c Intervention adherence and tolerability (How was this assessed?)**: Not applicable given the short-term and targeted nature of the intervention, which precluded the need for long-term adherence or tolerability assessment.
5. **10d Adverse and unanticipated events**: Not applicable as the treatment did not yield any adverse effects or complications, and the patient experienced a favorable recovery.
6. **12 The patient should share their perspective in one to two paragraphs on the treatment(s) they received**: Not applicable as the patient's perspective was not obtained in the format required by the checklist, and the clinical emphasis was placed on diagnostic and therapeutic details rather than on a narrative account.
